# Emotional Dysregulation and Altered Reward Processing in Self-Harm

**DOI:** 10.1192/bjo.2022.261

**Published:** 2022-06-20

**Authors:** Emre Yavuz, Rachel Rodrigues, Martina Di Simplicio

**Affiliations:** Imperial College London, London, United Kingdom

## Abstract

**Aims:**

Self-Harm (SH) is defined as “any act of self-injury or poisoning carried out by a person irrespective of their motivation”. SH increases the risk of adverse outcomes including suicide attempts, necessitating early intervention. The most widely reported reason for SH is to relieve negative affect (NA), with NA precipitating SH engagement. SH participants show altered reward processing, particularly reward hypersensitivity. NA could trigger reward hypersensitivity and therefore SH engagement. However, the interaction between NA and reward processing in SH remains unclear.

Aim: To investigate whether those who SH show differences in processing SH stimuli compared to healthy controls (HCs) following NA induction.

Hypothesis: NA induction will result in SH participants having significantly shorter reaction latency (RL) and significantly greater reaction accuracy (RA) in the SH condition of the Incentive Delay task (IDT) than HCs.

**Methods:**

16–25-year-old SH (n = 35) and HC (n = 20) participants were recruited on social media. Participants completed the Trier Social Stress Test, to induce NA, followed by the IDT. In the latter, participants were cued to respond to a target as quickly as possible, and on responding were shown images of either a SH act (SH condition), people socializing (social condition) or money (monetary condition), where each condition had control trials where a neutral image was shown, which participants also had to respond to (SH neutral, social neutral and monetary neutral conditions respectively). RA was the percentage of IDT trials in which participants responded within the target's presentation time. RL in the IDT was the time (seconds) between the target appearance and the participant's response.

**Results:**

A linear mixed effects model showed no significant main effect of group on RL (SH vs HC), condition (Social, SH or Monetary) or group x condition interaction (p > 0.05). There was a significant main effect of condition on RA (p < 0.05) but not group or group x condition interaction (p > 0.05). Past-week SH frequency and RA were significantly and positively correlated in social, social neutral and monetary conditions (p < 0.05).

**Conclusion:**

Overall, there was a non-significant effect of NA on reward processing. However, as greater past-week SH frequency was significantly associated with greater RA, understanding how reward processing and NA interact in SH can provide greater insight into its triggers. Given this study's limited sample size and cross-sectional nature, future studies should investigate how NA and reward processing interact longitudinally and in larger samples to understand how SH can be reduced.

